# An LMS Programming Scheme and Floating-Gate Technology Enabled Trimmer-Less and Low Voltage Flame Detection Sensor

**DOI:** 10.3390/s17061387

**Published:** 2017-06-14

**Authors:** Juan Carlos Iglesias-Rojas, Felipe Gomez-Castañeda, Jose Antonio Moreno-Cadenas

**Affiliations:** 1 Department of Communications and Electronics, National Polytechnic Institute, Mexico City 07738, Mexico; 2 Department of Electrical Engineering, Center for Research and Advanced Studies of the National Polytechnic Institute, Mexico City 07360, Mexico; fgomez@cinvestav.mx (F.G.); jmoreno@cinvestav.mx (J.A.M.)

**Keywords:** flame detector sensor, CMOS analog integrated circuits, field programmable gate arrays, operational amplifiers, LMS, industrial heaters

## Abstract

In this paper, a Least Mean Square (LMS) programming scheme is used to set the offset voltage of two operational amplifiers that were built using floating-gate transistors, enabling a 0.95 V_RMS_ trimmer-less flame detection sensor. The programming scheme is capable of setting the offset voltage over a wide range of values by means of electron injection. The flame detection sensor consists of two programmable offset operational amplifiers; the first amplifier serves as a 26 μV offset voltage follower, whereas the second amplifier acts as a programmable trimmer-less voltage comparator. Both amplifiers form the proposed sensor, whose principle of functionality is based on the detection of the electrical changes produced by the flame ionization. The experimental results show that it is possible to measure the presence of a flame accurately after programming the amplifiers with a maximum of 35 LMS-algorithm iterations. Current commercial flame detectors are mainly used in absorption refrigerators and large industrial gas heaters, where a high voltage AC source and several mechanical trimmings are used in order to accurately measure the presence of the flame.

## 1. Introduction

Flame detector sensors are widely used for industrial applications such as absorption refrigerators and large gas heaters. Some methods to detect a flame were already published. The methods described in [1,2,3,4], utilize an image sensor and image signal processing to determine the presence of flames. Not only is this method expensive, but also complex. In [5], a silicon micro-structure to detect micro-flames is presented, but, due to the small size of the structure, it is difficult to implement it in large industrial applications. In [6,7] an ultraviolet (UV) sensor is used to detect a flame. The UV sensor has low reliability because the phototube applied as a detection device can easily become detached; furthermore, the UV sensor is temperature sensitive, so complex mechanical parts are needed.

In this paper, the flame ionization principle was used to measure the presence of flames. This principle states that a single flame generates free carriers that bring about current conduction [8]. The flame detection sensor designed for this work exploits this principle to accurately measure the presence of the flame.

Unfortunately, the flame ionization principle requires a high AC voltage to deliver reliable flame detection [8,9,10]. The flame detection principle consists of a pure AC voltage applied though the flame using a metal rod; the flame acts as a low forward current diode [8], so, a very small DC voltage component is present at the rod. Therefore, the aim of the flame detector sensor circuit is to detect a very small DC voltage component mounted on a large AC voltage.

According to [8], the AC voltage source that feeds the flame through the rod, should provide at least 5 V (more than 100 V_RMS_ is recommended) to obtain a relative high DC component mounted on the large AC signal. This requirement is based on the assumption that common circuits, e.g., Bipolar Junction Transistors (BJT), common commercial operational amplifiers, etc. will be used. Common commercial circuits that can handle high input voltages are frequently high offset voltage devices. The offset voltage is very important for this application because the flame produces a very small DC component that in most cases is lower than the input referred offset voltage of the amplifiers used, resulting in detection failure.

In this paper, an offset-cancelled operational amplifier and a trimmer-less field programmable analog comparator are used to detect the very small DC component produced by the flame; furthermore, the circuit design was implemented on chip so that typical industrial requirements such as robustness and reliability are met.

To be compliant with the offset voltage requirements, a Programmable Offset Operational Amplifier (POOA) was used [11,12]. The POOA is the heart of the proposed flame detection sensor due to its capability of setting its offset voltage by means of programming. The POOA can be used for different applications, e.g., a precision amplifier (an amplifier whose offset voltage σ, is lower than 100 μV), or a voltage comparator, where σ is the decision level.

A CMOS technology chip of 1.2 μm with an oxide thickness of 316 Å was used to implement the POOAs, however, submicron technologies such as 0.35 μm, 0.25 μm, and 0.18 μm are recommended since these fabrication technologies present better performance and reliability [13].

The POOA is based on Floating Gate Metal Oxide (FGMOS) field effect transistors that should be programmed by means of hot electron injection and electron tunneling. FGMOS applications that utilize hot electron injection and electron tunneling require a fast and accurate programming scheme in order to improve reliability and yield efficiency. A typical programming scheme is used in [14,15], where the source-drain voltage (*V_SD_*) is modulated for controlling the injected current into the floating gate. This scheme presents fast and accurate programming, but the circuit implementation is complex.

The proposed programming scheme utilizes the least mean square (LMS) algorithm to control the frequency of the injection pulses in order to set accurately the floating-gate voltage in FGMOS transistors. Due to the ease of LMS computational implementation and the use of injection pulses instead of *V_SD_* modulation, this programming scheme dramatically reduces the circuit implementation complexity, therefore the LMS algorithm was used for setting the offset voltage of an operational amplifier over a wide range of values.

The experimental results show that it is possible to measure the presence of a flame using a 60 Hz AC input signal of just 0.95 V_RMS_. These results were tested after programming two POOAs. The POOAs were programmed using an LMS algorithm implemented into a Field Programmable Gate Array (FPGA). The main contributions of this paper can be summarized as follows:

1. A low voltage flame detector sensor based on the flame ionization principle.
2. A trimmer-less voltage comparator.
3. A programming scheme that is based on the LMS algorithm.
4. An easy circuit implementation of the LMS algorithm.
5. A programming circuit that utilizes injection pulses instead of *V_SD_* modulation.

The organization of the paper is as follows: first, in Section 2, the flame ionization principle and the flame detection sensor scheme are presented; circuit analysis is also discussed. In Section 3, a detailed description of the POOA design is shown. In Section 4, details of the LMS programming scheme as well as the FPGA implementation of the LMS algorithm is presented. The circuit design of the flame detecting circuit based on two POOAs is presented in Section 5, and the experimental results of the LMS programming performance and the measurement of the flame presence are shown in Section 6. Finally, a conclusion of the results obtained is presented in Section 7.

## 2. The Flame Detection Sensor

### 2.1. The Flame Ionization Principle

The flame ionization principle is described and studied in depth in [8]. This principle is used not only to detect the flame presence, but also to characterize the flame behavior [16]. According to [8], all flames conduct electrical current by means of the free ions generated by the flames themselves. The most accepted argument of this principle is explained by the chemical reaction shown in Equation (1):

(1) $$CH+O\overset{\Delta }{\to }CHO^{+}+e^{-}$$

**Figure 1:**
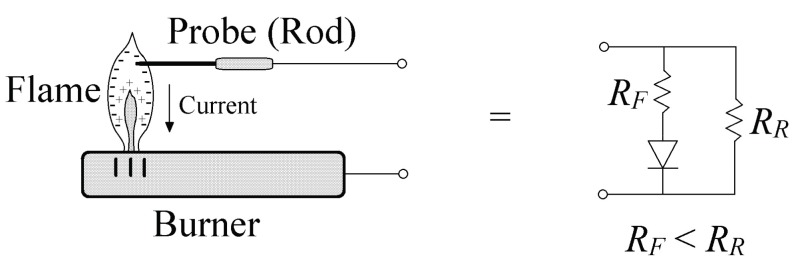
The equivalent circuit of a flame ionization measurement system. *R_F_* and *R_R_* are the forward and reverse flame resistances, respectively. The forward resistance is always lower than the reverse resistance, no matter the AC voltage applied to the flame.

In theory, the flame conducts electrical current in one direction, i.e., it acts as a diode. In Figure 1, the equivalent circuit of an active flame is presented. The equivalent circuit is formed by a diode and two resistors; the diode is supposed to be ideal, whereas the resistors are of different values that fulfill the rule: *R_F_* < *R_R_*. *R_F_* and *R_R_* are the forward and reverse flame resistances, respectively.

In reference [8], a deep study of the flame resistances was made, concluding that the *R_R_*/*R_F_* relation depends on the rod (flame test probe) position and the AC voltage applied to the flame. For voltages lower than 5 V, the *R_R_*/*R_F_* relation is a little bit higher than the unit. Therefore, the flame behaves as a non-ideal diode whose forward and reverse currents are very similar. The forward current (from the rod to the burner) is always higher than the reverse current. The rectification property of the flame has been widely used in flame detectors due to its reliability and robustness [9,10].

### 2.2. The Flame Detection Sensor Scheme

**Figure 2:**
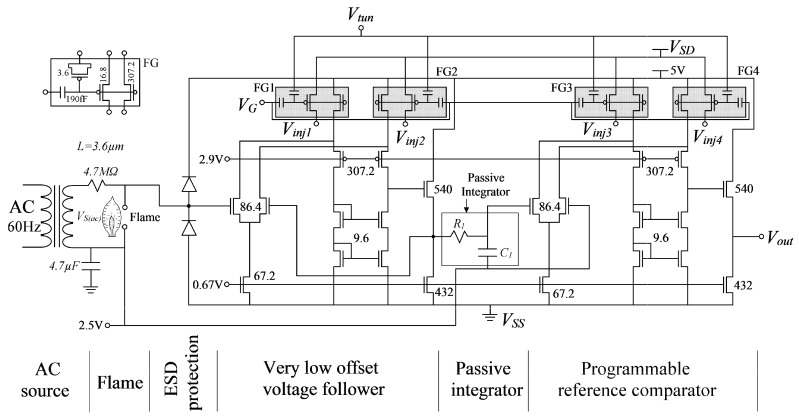
The flame detecting sensor using CMOS technology and Floating Gate Transistors. The AC voltage is applied through the flame by means of a metal rod and a burner. The two Programmable Offset Operational Amplifiers and the integrator’s network are clearly shown.

The complete design of the flame detecting sensor using two POOAs is shown in Figure 2, where the first POOA was configured as an offset-cancelled voltage follower, and the second POOA was configured as a voltage comparator. A 60 Hz AC signal is applied to the flame through a 4.7 MΩ resistor, so that the rectifying property of the flame produces a DC component while the flame is present. The DC component is separated of the 60 Hz AC signal by means of a passive integrator circuit formed by *R*_1_ and *C*_1_. Because the flame ionization measurement system is high impedance and the DC component produced by the flame would be down to a few millivolts, an offset-cancelled voltage follower is needed. The passive integrator circuit formed by *R*_1_ and *C*_1_, dramatically reduces the AC signal, whereas the DC component will pass through it with almost no attenuation. The integrator’s output signal is then compared to a decision level given by the second POOA in order to detect the presence of the flame. The second POOA is configured as a voltage comparator, whose offset voltage *σ* is the decision level. While the flame is active, a DC voltage with a small 60 Hz ripple is presented at the non-inverting comparator’s input. Since the DC component of this signal is greater than the comparator’s decision level, the comparator’s output will become logically high.

## 3. The Programmable Offset Operational Amplifier

### 3.1. The POOA Design

**Figure 3:**
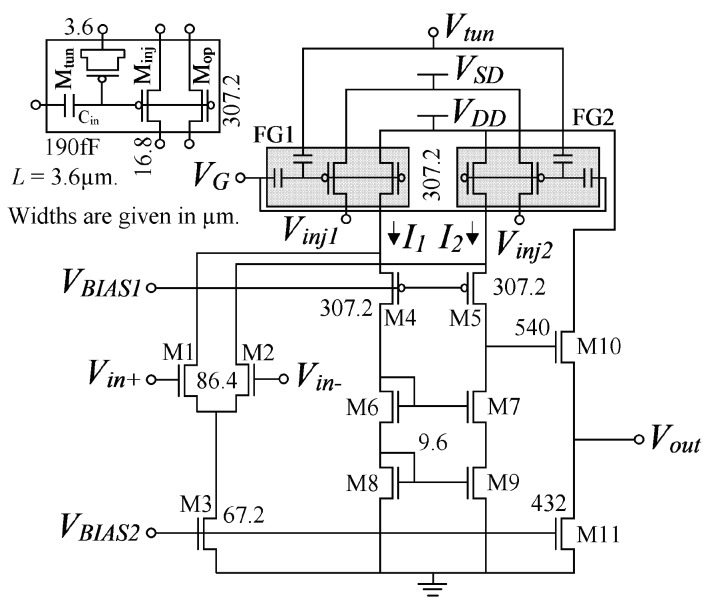
The POOA circuit design. FG1 and FG2 are indirect programming floating-gate structures, M1 and M2 are the input differential pair transistors, M4–M9 are the folded-cascode transistors, and M10–M11 are the output buffer transistors.

The circuit design of the POOA is presented in Figure 3. FG1 and FG2 are indirect programming floating-gate structures [15], M1 and M2 are the input differential pair transistors, M3 is the input differential pair bias current source, M4–M9 are the folded-cascode transistors, and M10–M11 are the output buffer transistors.

The circuit design procedure (including transistors M_op1_ and M_op2_) is presented in [17], and the simulation model for circuit design is presented in [18]. Adding a tunneling transistor (M_tun_), an injection transistor (M_inj_), and a control gate capacitor (*C_in_*), to each tail of the folded-cascode amplifier, forms a POOA. The length (*L*) of all transistors is 3.6 μm, whereas the widths (*W*) were obtained from the geometry relations (*S* = *W*/*L*) [17]. The final widths were modified by means of PSpice simulations in order to increase the POOA’s phase margin [11].

**Figure 4:**
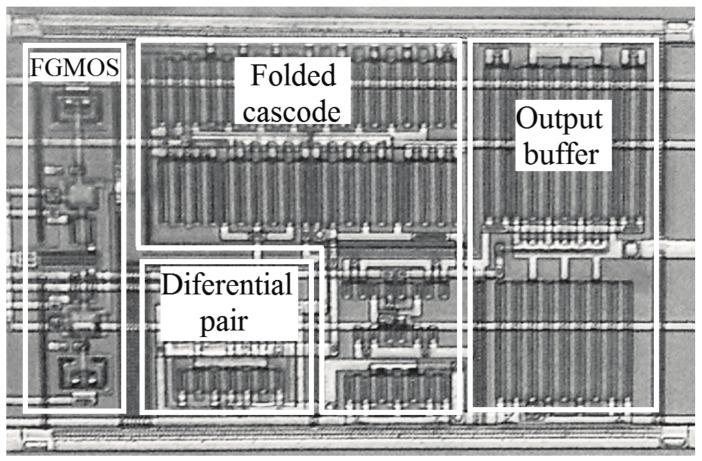
Microphotograph of the POOA. The final layout is 400 μm × 245 μm and requires about 15.6% more die area than a regular folded-cascode operational amplifier.

The tunneling voltage (*V_tun_*) is equal to *V_SS_* during normal operation and programming process, and should be increased up to 27 V for tunneling. The injection voltages (*V_inj1_* and *V_inj2_*) are equal to *V_DD_* during normal operation and tunneling process and equal to *V_SS_* during programming. The programming voltage (*V_SD_*) is equal to *V_DD_* during normal operation and tunneling process and equal to 7.5 V during programming. The programming voltage should be increased up to 8 V to bring on the injection current, and consequently, increase the adaptation rate (*ξ*) explained in the next section. The main POOA’s electrical parameters obtained experimentally are shown in Table 1 and its microphotograph is shown in Figure 4.

### 3.2. The POOA as A Precision Amplifier

In practice, any operational amplifier presents an offset voltage due to mismatch. The offset voltage can be reduced by adding floating-gate structures into a folded-cascode topology. From Figure 3, it is observed that the tail currents *I*_1_ and *I*_2_, depend on the floating-gate voltages of FG1 and FG2, respectively. If it is assumed that the floating-gate voltages (*V_FG1_* and *V_FG2_*) are equal, and the input voltages (*V_in+_* and *V_in−_*) are also equal and within the input range voltage interval, the currents *I*_1_ and *I*_2_ are equal in theory. Again, due to mismatch, the tail currents of the folded-cascode are different in practice, causing an offset voltage at the amplifier’s output. Because the tail currents of the folded-cascode can be controlled by means of the floating-gate voltages, and the floating-gate voltages depend on the charge stored in them, the tail currents can be programmed by means of electron injection and tunneling. The electron tunneling removes electrons from the floating gate, increasing the floating-gate voltage; to the contrary, electron injection inserts electrons into the floating gate, decreasing the floating-gate voltage. Equation (2) describes the floating-gate voltage at FG1 and FG2:

(2) $$V_{FG}=\frac{C_{in}V_{in}-Q_{FG}}{C_{T}}$$

where *Q_FG_* is the total electron charge stored in the floating gate, and *C_T_* is the total capacitance seen by the floating gate. The capacitances *C_in_* and *C_T_* and the voltage *V_in_* are constant, so, the floating-gate voltage only depends on the total charge stored in it. Equation (2) describes the floating-gate voltage dependence of the total floating-gate charge. Electron tunneling increases the floating-gate voltage, whereas electron injection decreases it, which means that electron tunneling decreases the tail current, whereas electron injection increases it.

## 4. LMS Programming Scheme

### 4.1. The LMS Algorithm

The LMS algorithm was presented for the first time by Widrow and Hoff in 1959. The LMS is a stochastic gradient descent algorithm that iterates the weight of each tap of a transversal filter in direction of the instantaneous gradient of the square error signal [19]. Despite the fact that the LMS algorithm is very complex in mathematical terms, it is easy to implement it in computational terms. Equation (3) shows the simplicity of the LMS algorithm update rule:

(3) $$\omega_{i}(n+1)=\omega_{i}(n)+\xi e(n)x(n)$$

where *ω_i_* is the weight of the tap *i*, *n* is the iteration number, *x*(*n*) is the input signal, *e*(*n*) is the error signal, and *ξ* is the adaptation rate. Despite the LMS algorithm simplicity, it has a fast and stable convergence compared to other adaptive algorithms such as the Hybrid RLS-NLMS [20].

The LMS algorithm has been proven to be fast and robust as described in [21]. These characteristics are suitable for industrial applications such as measurement equipment and sensor calibration.

### 4.2. Programming Scheme Based on LMS Algorithm

**Figure 5:**
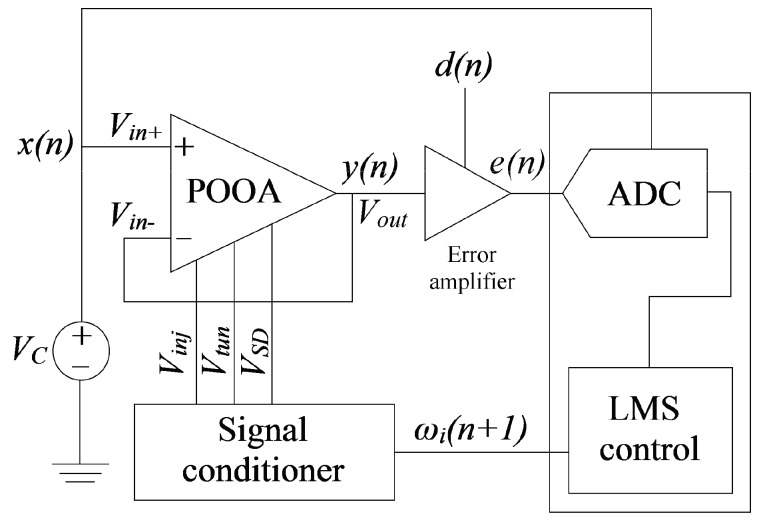
The POOA’s programming scheme based on LMS algorithm. The POOA is connected as a voltage follower in order to read the offset voltage directly. Because the offset voltage could be too small, an error amplifier is used. The ADC digitizes the error signal, so that the LMS control block is able to process it.

The programming scheme based on LMS algorithm is shown in Figure 5. The POOA is connected as a voltage follower with an input voltage equals to the common mode voltage (*V_C_* = 2.5 V).

The offset voltage can be obtained by subtracting the POOA’s output voltage (*V_out_*) from the input common mode voltage. *V_C_* is also used as the input signal *x*(*n*). The desired signal (*d*(*n*)) is obtained from Equation (4):

(4) $$d(n)=V_{C}-\sigma$$

Equation (4) is the starting point of the programming scheme. At this point, the desired offset voltage is selected in order to obtain *d*(*n*) with a common mode voltage *V_C_* = 2.5V, e.g., if an offset voltage of +5 mV is desired, *d*(*n*) yields 2.495 V. For offset cancellation (*σ* = *0*), *d*(*n*) = *V_C_*. *V_out_* is used by the error amplifier to obtain the error signal. The error amplifier is a commercial non-inverting linear operational amplifier with an input referred offset voltage of 15 μV whose commercial manufactured number is OP177. The error voltage, according to Figure 5 is given by:

(5) $$e(n)=\left(y(n)-d(n)\right)A_{Verror}+V_{C}$$

where *A_Verror_* is the error amplifier’s gain. Equation (5) shows that the error signal is a signed value. If *y*(*n*) is greater than *d*(*n*), the error signal is positive, to the contrary, if *y*(*n*) is lower than *d*(*n*), the error signal is negative. Because the ADC cannot read negative values, a common mode voltage (*V_C_*) is added, so that the ADC reads a value of ‘0’ when *e*(*n*) = *V_C_*. The error signal values greater than *V_C_* are positive values, i.e., the most significant bit (MSB) of the ADC represents the error signal sign. If MSB = ‘1’, the error signal is positive, otherwise, the error signal is negative. The lower seven bits of the ADC output represent the magnitude of the error signal.

The digitalized-signed-error signal is processed by the LMS control block in order to generate the programming pulses. Because the amplitude of the programming pulses at the FPGA terminals is 2.5 V and the amplitude of the pulses in *V_SD_* to enable electron injection is approximately 7.5 V, a signal conditioner is needed. The signal conditioner is also needed to condition the 27 V-tunneling pulse in amplitude.

The weight *ω*(*n*) is stored into the internal floating gate of the POOA. The injection pulses generated by the LMS algorithm update the floating-gate voltage, i.e., the weight update *ω*(*n* + *1*) is updated according to the LMS learning rule. This programming scheme was previously used to cancel the offset voltage only [10], but not to program the offset voltage over a wide range of values.

### 4.3. FPGA Implementation of the Programming Scheme

The LMS algorithm can be implemented in many different ways. In [22], the LMS algorithm was implemented into a PIC microcontroller to program a memory cell by means of electron tunneling and injection. This scheme is highly clock-speed limited due to the microcontroller architecture. In [23], the LMS algorithm was implemented into an FPGA to obtain the TAP weights of a finite impulse response (FIR) filter, reducing the clock-speed limitation. Both programming schemes are quite complex to implement due to the high speed and high voltage signal conditioner used for the tunneling pulses.

The presented programming scheme utilizes not only an FPGA for implementing the LMS algorithm so that the clock speed does not represent a serious limitation, but also electron injection and indirect programming to avoid high voltages needed for electron tunneling. We chose an FPGA to implement the LMS algorithm, because it presents advance features that are suitable for industrial applications [24].

**Figure 6:**
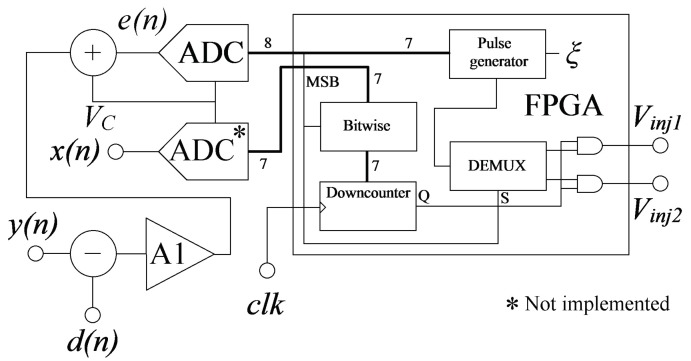
The FPGA implementation of the LMS algorithm. The substracter, the adder, and the low-offset error amplifier (A1) are used to obtain the signed error signal. The ADCs are used to digitize *e*(*n*) and *x*(*n*). The LMS algorithm is implemented into the FPGA.

The LMS algorithm was actually implemented using a mixed-signal circuit. The analog part consists of an adder, a subtractor, and a low-offset error amplifier (A1), the digital part consists of an FPGA that contains the pulse generators, and the mixed-signal part consists of an 8-bit ADC. The LMS algorithm implemented into the FPGA is a modified version of the functional link artificial neural network presented in [25]. In Figure 6, the FPGA implementation of the LMS-algorithm can be seen.

Equation (5) is implemented in the analog part (the subtractor, the adder and the low-offset error amplifier). These analog circuits form the error amplifier of Figure 5. In Figure 6, there are two ADCs, the first one is a single 8-bit ADC used to digitize the error signal, whereas the second one is a single 7-bit ADC used to digitize the input signal *x*(*n*). Because *x*(*n*) is constant (2.5 V), the second ADC was not physically implemented, instead, the ADC was replaced by a digital constant value (the hexadecimal 0 × 7F).

The digitized error signal is taken to the FPGA to be processed. The lower seven bits of the error signal are connected to the pulse generator. The pulse generator outputs a number of pulses that is equal to the magnitude of the error signal during a constant time interval (*T_PG_*). The pulse duration (*T_PON_*) is 100 μs, and the time interval *T_PG_* is always 25.6 ms. The greater the error, the greater the number of pulses at the output of the pulse generator, thus, increasing the injection current.

The total low state time (*T_OFF_*) within the *T_PG_* interval can be obtained from Equation (6):

(6) $$T_{OFF}=T_{PG}-e(n)T_{PS}$$

The adaptation rate (*ξ*) is consequently controlled by the pulse duration (*T_PON_*). On the other hand, the input signal is bitwised (XORed) according to the error signal sign in order to obtain the error magnitude. This technique for obtaining the error magnitude is used by the pulse generator too. The error magnitude is then used to pre-load a 7-bit downcounter to open a time window for the generator pulses. The MSB is used to obtain the error sign and select the injection output which will output the programming pulses.

### 4.4. The POOA as A Voltage Comparator

In [11], the Programmable Offset Operational Amplifier was used as a precision amplifier, reducing the input referred offset voltage down to 25 μV. In this work, the same POOA not only was used as a precision amplifier, but also as a Programmable Reference Voltage Comparator. The main idea was to use the current programming scheme to set an offset voltage different than zero. To achieve this, a *d*(*n*) ≠ 0 should be chosen. The value of the signal *d*(*n*) is obtained from Equation (5). Let us assume a hypothetical situation where a POOA has an offset voltage of −4 mV, the POOA is connected as indicated in Figure 5, the error voltage gain (*A_Verror_*) is equal to 100, and the desired offset voltage is +15 mV; *d*(*n*) is 2.485V according to Equation (4) and the error voltage is 4.4 V at the beginning of the programming process according to Equation (5).

**Figure 7:**
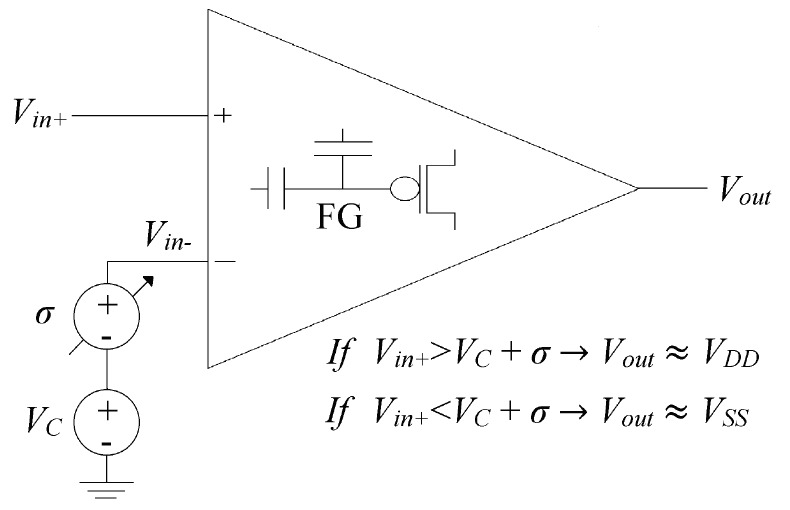
The POOA as a voltage comparator. To implement a voltage comparator, the POOA should be connected in an open loop topology and the inverted input (*V_in−_*) should be taken to *V_C_*. The input referred offset voltage behaves as a DC power supply inserted into the *V_in+_* path, so that the comparator decision level depends on the offset voltage.

The ADC will output a positive digital value (*e*(*n*) > *V_C_*), so that the lower seven bits of the digitized error signal will not be bitwised to obtain the error magnitude. The pulses at the pulse generator output will be processed by a logical AND during the time window generated by the downcounter in order to obtain the injection pulses at the injection terminal V_inj1_ (MSB = ‘1’), thus increasing the tail current *I*_1_, and consequently, decreasing the POOA’s output voltage. The error voltage is reduced on each LMS-algorithm iteration until it becomes zero, when this happens, the POOA’s output voltage is equal to 2.485 V, which means that an offset voltage of +15 mV has been programmed. The total offset voltage deviation (Δ_σ_) was +19 mV. The maximum offset voltage deviation that can be programmed depends on the ADC input range (*IR_ADC_*) and the error amplifier’s gain (*A_Verror_*), e.g., for an ADC with an input range of 0V to 5 V and an error amplifier’s gain of 100, the maximum voltage deviation (Δ_σmax_) is ±50 mV according to Equation (7). In Figure 7, a voltage comparator implemented by means of a POOA is shown:

(7) $$\Delta_{\sigma \max}=\frac{IR_{ADC}}{A_{Verror}}$$

## 5. Quantitative Analysis of the Flame Detection Sensor

### AC/DC Signal Behavior

**Figure 8:**
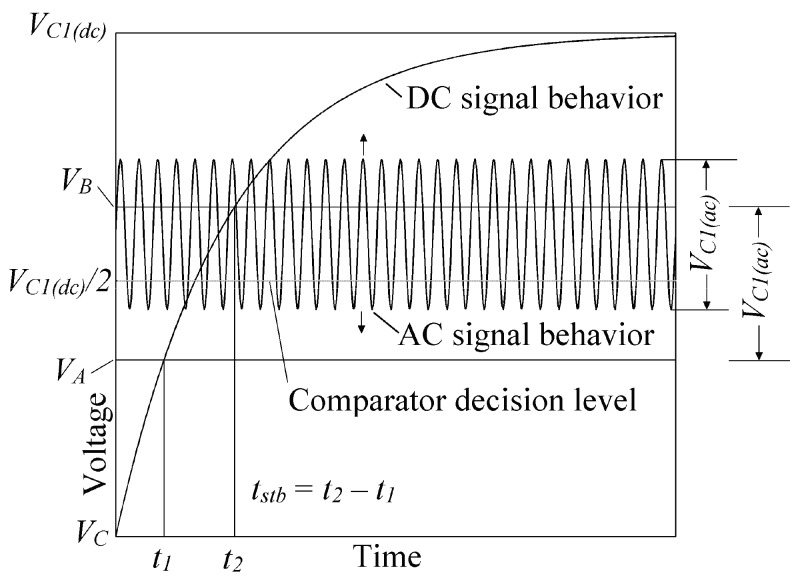
The AC and DC signal behavior of the flame detecting circuit. The AC signal is attenuated by the passive integrator circuit, whereas the DC signal follows an exponential behavior. The comparator’s decision level is *V_C_*_1(*dc*)_/2. The comparator’s decision level was chosen *V_C_*_1(*dc*)_/2 in order to reduce the noise margin.

In order to understand the actual behavior of the flame detecting circuit, a quantitative analysis should be done by studying the AC and DC signal behavior. In Figure 8, a graphical description of the AC and DC behavior of the signal is presented.

Let us call *V_S_*_(*ac*)_ the peak to peak AC voltage at the transformer’s output terminals (see Figure 2), *V_C_*_1(*ac*)_ the peak to peak AC component voltage at *C*_1_ terminals, and *V_C_*_1(*dc*)_ the DC voltage component produced by the rectification effect of the flame at *C*_1_ terminals.

From Figure 2, when no flame is present at the rod, a pure 60 Hz AC signal is present at the input of the very low offset voltage follower. Since the input referred offset voltage (*σ*) of the voltage follower is many times lower (−26 μV) than *V_C_*_1(*ac*)_ (few mili-volts), the signal is practically unaffected. The pure AC signal then feeds a passive integrator in order to attenuate the AC component and extract the DC component. In this case, the DC component is practically zero, so, a very small pure AC signal will be present at the input of the programmable reference comparator. The comparator is already programmed with a decision level greater than the peak voltage of the AC component at the passive integrator output, so, when no flame is present at the rod, the comparator will output a logical zero, that is, a voltage close to *V_SS_*.

To the contrary, when a flame is present at the rod, the AC signal present at the input of the very low offset voltage follower contents a small DC component due to the rectification properties of the flame. Again, since the input referred offset voltage (*σ*) of the voltage follower is many times lower than *V_C_*_1(*ac*)_, the signal is practically unaffected. Since the signal now contains a DC component, this appears at the passive integrator’s output. The DC component *V_C_*_1(*dc*)_ at the input of the programmable offset comparator is higher than the comparators decision level, so, when the flame is present at the rod, a logical one, i.e., an output voltage close to 3.3 V (according to the output range of the POOA) will appear at the comparator’s output.

When the flame is present at the rod, a small AC component will appear at the input of the programmable comparator due to the passive integrator effect, whereas the DC component will start from zero volts to a maximum voltage *V_C_*_1(*dc*)_ that depends on the flame properties and rod position [8]. The DC component will increase its voltage according to the well-known capacitor charge equation (Figure 8).

It is well know that a diode (in this case the flame) has an I/V behavior that is inherently non-linear. Since the fundamental component of the actual signal is the greatest in amplitude, an approximation considering just this 60 Hz component was done. Thus, the peak to peak AC voltage at *C*_1_ terminals is obtained from (8):

(8) $$V_{C1(ac)}=\frac{V_{S(ac)}X_{C1}}{R_{1}+X_{C1}}=\frac{V_{S(ac)}}{1+2\pi f\tau }$$

where *f* is the line frequency and *τ* is the time constant that is equal to *R*_1_*C*_1_. The DC signal behavior is obtained from the capacitor charge equation, therefore the instantaneous voltage at *C*_1_ terminals yields:

(9) $$V_{C1}(t)=V_{C1(dc)}\left(1-\exp(t/\tau \right)$$

As shown in Figure 8, the comparator’s decision level is *V_C_*_1(*dc*)_/2 due to the reduction of noise margin. Let’s assume that a 60 Hz signal with a peak to peak voltage *V_C_*_1(*ac*)_ lower than the comparator’s decision level *V_C_*_1(*dc*)_/2. At the moment the flame is present at the rod, the signal start to increase its DC component exponentially, when the DC component is equal to *V_A_* = *V_C_*_1(*dc*)_/2 − *V_C_*_1(*ac*)_/2, the positive peak of the signal reaches the comparator’s decision level, thus, a rectangular wave starts to appear at the comparator’s output. The rectangular wave continues appearing at the comparator’s output until the DC component *V_C_*_1_(*t*) is equal to *V_B_ = V_C_*_1(*dc*)_/2 *− V_C_*_1(*ac*)_/2, i.e., the negative peak of the signal just leaves the comparator’s decision level. From Figure 8, the rectangular wave at the comparator’s output starts at time *t*_1_ and ends at time *t*_2_.

The time interval in which the comparator’s output is pulsing is called stabilization time (*t_stb_*). From Figure 8, the stabilization time is *t*_2_ − *t*_1_. *t*_1_ and *t*_2_ are obtained using Equations (10) and (11).

(10) $$t_{1}=-\tau \ln\left(\frac{\left(V_{C1(dc)}/2\right)+\left(V_{C1(ac)}/2\right)}{V_{C1(dc)}}\right)$$

(11) $$t_{1}=-\tau \ln\left(\frac{\left(V_{C1(dc)}/2\right)-\left(V_{C1(ac)}/2\right)}{V_{C1(dc)}}\right)$$

(12) $$\begin{array}{l} t_{stb}=t_{2}-t_{1}\\ t_{stb}=\tau \ln\left[\frac{\left(V_{C1(dc)}+V_{C1(ac)}\right)/2V_{C1(dc)}}{\left(V_{C1(dc)}-V_{C1(ac)}\right)/2V_{C1(dc)}}\right]\\ t_{stb}=\tau \ln\left(\frac{V_{C1(dc)}+V_{C1(ac)}}{V_{C1(dc)}-V_{C1(ac)}}\right) \end{array}$$

**Figure 9:**
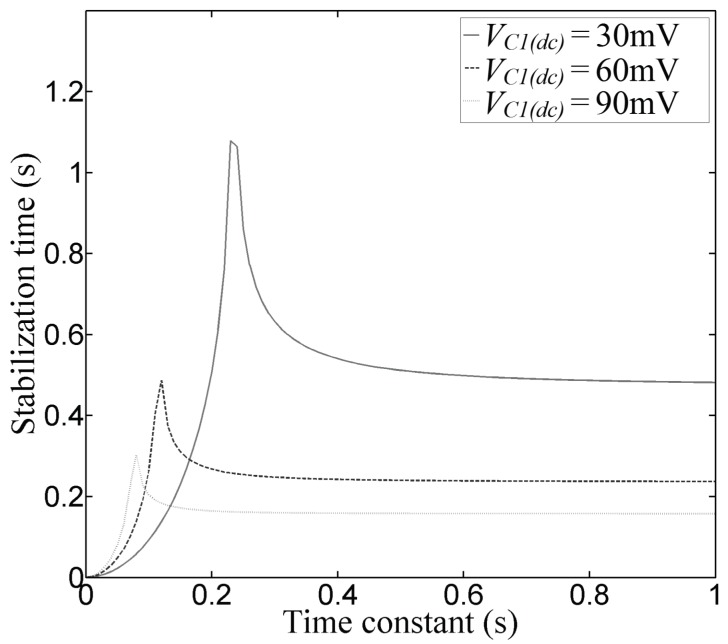
Stabilization time vs time constant plot. The stabilization time reaches its maximum value when the time constant is equal to *τ*(*min*). For all values of *τ* lower than *τ*(*min*), the stabilization time results in an imaginary value, it means that an unstable state in which the system (flame detector sensor) outputs a rectangular wave always. For large τ values, the stabilization time remains practically constant.

Equation (12) shows that *t_stb_* depends mainly on the time constant, this is not true at all due to *V_C_*_1(*ac*)_ depends on the time constant too. In Figure 9, the stabilization time vs the time constant is plotted for various DC voltages (*V_C_*_1(*dc*)_) and a *V_S_*_(*ac*)_ voltage equals to 2.68 V (0.95 V_RMS_). The values of R_1_ and C_1_ are obtained from *τ* as seen ahead.

In order to prevent instability, the maximum value of *V_C_*_1(*ac*)_ should be lower than *V_C_*_1(*dc*)_, otherwise, a continuous rectangular signal will appear at the comparator’s output even when the flame is not present. To obtain the minimum time constant value, we substitute *V_C_*_1(*ac*)_ by *V_C_*_1(*dc*)_ in (8) and solve for *τ*.

(13) $$\tau_{\left(\min\right)}=\frac{V_{S(ac)}-V_{C1(dc)}}{2\pi fV_{C1(dc)}}$$

As seen in Figure 9, the stabilization time increases dramatically when *τ* is near to *τ*(*min*). The maximum value of *t_stb_* is reached when *τ* equals *τ*(*min*). The stabilization time depends basically on the DC signal *V_C_*_1(*dc*)_, therefore, *t_stb_* is reduced in practice, by moving the rod to a right angle [8].

As mention above, in order to reduce the noise margin, the comparator’s decision level should be select based on the minimum *V_C_*_1(*dc*)_ voltage, in this case, 30 mV (obtained experimentally with the flame present). The optimal decision level is *V_C_*_1(*dc*)_/2, i.e., 15 mV. Thus a POAA with an offset voltage of +15 mV should be programmed. The values of *R*_1_ and *C*_1_ were obtained from (8) given a *V_S_*_(*ac*)_ = 2.68 V and *f* = 60 Hz. It was obtained: *R*_1_ = 100 kΩ and *C*_1_ = 5.6 μF.

## 6. Sensor Test

### 6.1. The LMS Programming Scheme Performance

**Figure 10:**
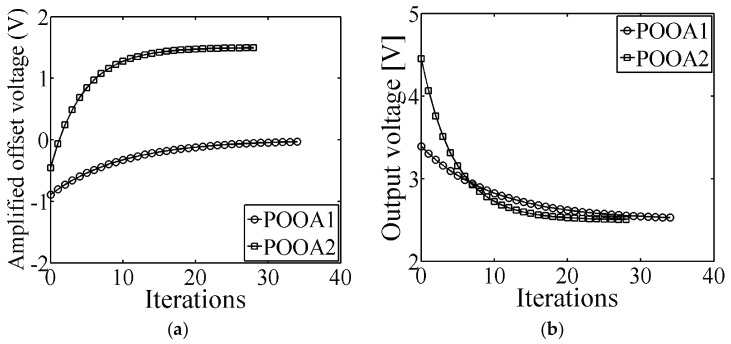
Test results of the programming performance. (**a**) The amplified offset voltage of both POOAs while the programming system is running. The error amplifier’s gain is 1000 for POOA1 and 100 for POOA2; (**b**) The LMS-algorithm error signal behavior.

In this paper, two POOAs were programmed by means of the proposed LMS programming scheme. The first POOA was programmed for offset removal; whereas the second one was programmed with a target offset voltage equals to +15 mV. In Figure 10a, the amplified offset voltage of both POOAs while the programming system is running is shown. The error amplifier’s gain is 1000 for POOA1 and 100 for POOA2.

The offset voltage is obtained by dividing the amplified offset voltage (*σ*) by the error amplifier’s gain (*A_Verror_*). The initial offset voltage of POOA1 was −890 μV and the final offset voltage was −26 μV, whereas the initial offset voltage of POOA2 was −4.53 mV and the final offset voltage was +15 mV. In Figure 10b, the error voltage of the LMS programming system is shown. The error signal is obtained from (5); when the term (*y*(*n*) − *d*(*n*)) converges to zero, the error amplifier outputs a value equals to *V_C_*. The adaptation rates are 1.9 and 3.8 for POOA1 and POOA2, respectively.

As it is shown in Figure 10a,b, the LMS algorithm has an asymptotic behavior, enabling a fast an accurate programming. This behavior is well described in theory in [26]. Table 2 summarizes the experimental results of the LMS-algorithm programming processes for POOA1 and POOA2.

### 6.2. Testing the Sensor

**Figure 11:**
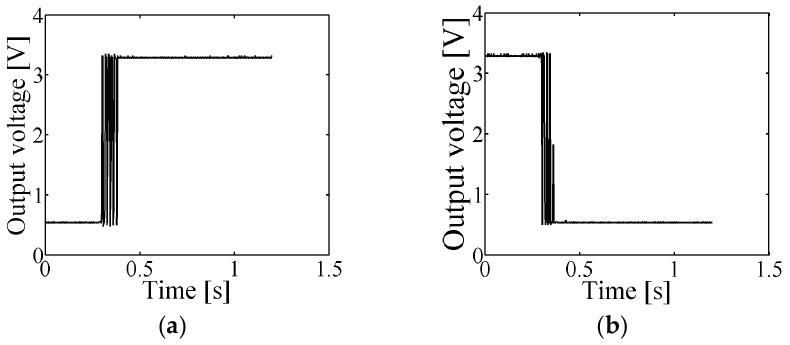
The flame detection sensor working. (**a**) The flame detector output signal when the flame is turned on. The stabilization time is around 100 ms; (**b**). The flame detector output signal when the flame is turned off. The stabilization time is around 60 ms.

The flame detection sensor was tested by turning on and turning off the flame. The time domain plot of the flame detector´s output when the flame is turned on is shown in Figure 11a. The stabilization time was around 100 ms. In Figure 11b, the time domain plot of the flame detector’s output when the flame was turned off is shown. The stabilization time was 60 ms. The logical zero was represented with an output voltage of 0.55 V, whereas the logical one was represented with an output voltage of 3.3 V. From Figure 11a,b, the flame was turned on and turned off at the time 0 (zero) respectively.

In theory, a stabilization time around 0.5 ms was expected, however, a shorter stabilization time was measured in practice; this is because the test probe was positioned in an optimized angle when the measurements were made.

**Figure 12:**
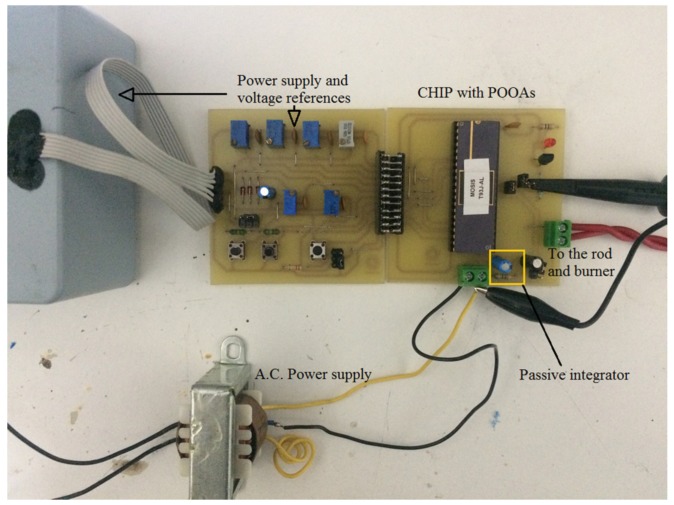
Picture of the entire flame detection sensor. The main parts of the system are clearly shown.

In Figure 12, the entire sensor system is presented. It is clearly shown the CHIP with the POOAs, the DC power supply and voltage references, the AC source to feed the flame, the off-chip passive integrator, and the board terminals to the rod and burner.

The proposed flame detection sensor is mainly used for industrial applications; it means that the sensor should work properly at various temperatures. In Table 3, the experimental results of the offset voltages of POOA1 and POOA2 after programming are shown. The offset voltage is the most critical parameter of the POOA to assure proper functionality of the sensor.

The experimental results of Table 3 show a maximum offset voltage deviation of −164 μV for POOA1 and −170 μV for POOA2 with respect to the theoretical values of 0V and +15 mV. Considering the effect of the offset voltage deviation of both POOA1 and POOA2, it is obtained a total offset voltage deviation of −334 μV, that is, the 2.2% of the programmed reference of the voltage comparator.

## 7. Conclusions

In this paper, a flame detection sensor based on the measurement of the electrical characteristics of the flame was presented. To achieve this, two programmable offset operational amplifiers (POOAs) were used. The POOAs were programmed using a reliable and robust LMS-algorithm programming scheme. The programming scheme can be employed as a good alternative to factory set the proposed sensor, avoiding the mechanical trimmers used in traditional flame detectors. The presented flame detection sensor is suitable not only for large industrial heaters, but also for low voltage flame detection applications such as home electronic boilers, where the power supply is a 3 V battery. The experimental results show some improvements compared with previous published works, for instance:

1. The AC voltage needed to feed the flame is considerably lower than the one used in commercial flame detectors.
2. The proposed flame detection sensor is trimmer-less, thus, increasing its reliability.
3. The proposed design is field-programmable.
4. The offset voltage deviation due to the temperature variation is negligible in terms of functionality.

Although the presented work yields satisfactory results, future work is needed to improve accuracy and reliability. The accuracy of the programming circuit can be increased by improving the ADCs resolution, whereas the reliability of the flame detector can be increased by using an AC current source instead of an AC voltage source as suggested in [9].

## Figures and Tables

**Table 1:** The main POOA’s electrical parameters.

Parameter ^1^	Magnitude	Unit
Open loop gain	65	dB
Bandwidth	235	kHz
Phase margin	60	dB
Slew rate	2	V/μs
Settling time	1550	ns
Output range	(*V_SS_* + 0.55) − (*V_DD_* − 1.7)	V
Common mode rejection ratio	65	dB
Power supply rejection ratio	70	dB
Input referred offset voltage	Programmable	V
Total power dissipation	7.2	mW

^1^ The above electrical parameters were measured at a room temperature of 27 °C and a supply voltage of 5 V.

**Table 2:** Summary of LMS programming processes.

Parameter	POOA1	POOA2
Initial offset voltage	−890 μV	−4.53 mV
Final offset voltage	−26 μV	+14.97 mV
Expected offset voltage	0 V	+15 mV
Initial error voltage	3.39 V	4.453 V
Final error voltage	2.526 V	2.503 V
Number of iterations	35	28
Adaptation rate	1.9	3.8
LMS clok frequency	5 kHz	5 kHz
Programming accuracy	-	99.8%
Total programming time ^1^	0.896 s	0.716 s

^1^ Not considering the erasing time (tunneling).

**Table 3:** Experimental results of the offset voltage of POOA1 and POOA2 at various temperatures.

Temperature	POOA1	POOA2
10 °C	+25 μV	+15.02 mV
27 °C	−26 μV	+14.97 mV
35 °C	−111 μV	+14.88 mV
50 °C	−164 μV	+14.83 mV

## References

[1] Qiu H., Chen F., Gao Y. Based on the CCD of the Boiler Flame Detection. Proceedings of the IEEE Third International Conference on Digital Manufacturing and Automation.

[2] Bae H., Kim S., Wang B.-H., Lee M., Harashima F. (2006). Flame Detection for the Steam Boiler Using Neural Network and Image Information in the Ulsan Steam Power Generation Plant. IEEE Trans. Ind. Electron..

[3] Zhang D., Wang Y. Real-Time Fire Detection Using Video Sequence Data. Proceedings of the IEEE 28th Chinese Control and Decision Conference.

[4] Chen H., Zhang X., Hong P., Hu H., Yin X. (2016). Recognition of the Temperature Condition of a Rotary Kiln Using Dynamic Features of A Series of Blurry Fame Images. IEEE Trans. Ind. Electron..

[5] Kuipers W.J., Müller J. Total Hydrocarbon Analysis with a Planar Micro Flame Ionization Detector. Proceedings of the IEEE 9th Sensors Conference.

[6] Cheong P., Chang K., Lai Y., Ho S., Sou I., Tam K. (2011). A ZigBee-Based Wireless Sensor Network Node for Ultraviolet Detection of Flame. IEEE Trans. Ind. Electron..

[7] Pauchard A.R., Manic D., Flanagan A., Besse P.A., Popovic R.S. (2000). A Method for Spark Rejection in Ultraviolet Flame Detectors. IEEE Trans. Ind. Electron..

[8] Möllberg A. (2005). Investigation of the Principle of Flame Rectification in Order to Improve Detection of the Propane Flame in Absorption Refrigerators. B.S. Thesis.

[9] Payne P.P., Schmidt S.E., Goppel K.P., King D.J., Tobin S.M., Fowler J.T. (2003). Constant Current Flame Ionization Circuit. U.S. Patent.

[10] Chian B., Anderson P.M., Nordberg T.J., Hill B. (2010). Flame Sensing System. U.S. Patent.

[11] Iglesias-Rojas J.C., Gomez-Castañeda G., Moreno-Cadenas J.A. Offset Reduction in Operational Amplifiers using Floating Gate Technology and LMS Algorithm. Proceedings of the IEEE 8th International Conference on Electrical Engineering Computing Science and Automatic Control.

[12] Iglesias-Rojas J.C., Gomez-Castañeda G., Moreno-Cadenas J.A. A Very Low Offset Voltage Operational Amplifier Using Floating-Gate Technology. Proceedings of the IEEE 20th International Conference on Electronics, Communication and Computer.

[13] Ma Y., Gilliland T., Wang B., Paulsen R., Pesaveento A., Wang H., Hoc N., Humes T., Diorio C. (2004). Reliability of pFET EEPROM With 70-Å Tunnel Oxide Manufactured in Generic Logic CMOS Processes. IEEE Trans. Device Mater. Reliab..

[14] Brink S., Hasler J., Wunderlich R. (2014). Adaptive Floating-Gate Circuit Enable Large-Scale FPAA. IEEE Trans. Very Large Scale Integr. Syst..

[15] Graham D.W., Farquhar E., Degnan B., Gordon C., Hasler P. (2007). Indirect Programming of Floating-Gate Transistor. IEEE Trans. Circuits Syst. I Reg. Pap..

[16] Li F., Xu L., Cao Z., Du M. A Chemi-Ionization Processing Approach for Characterizing Flame Flickering Behavior. Proceedings of the IEEE International Instrumentation and Measurement Technology Conference.

[17] Allen P.E., Holberg D.R. (2011). CMOS Analog Circuit Design.

[18] Rahimi K., Diorio C., Hernandez C., Brockhausen M.D. A simulation Model for Floating-Gate MOS Synapse Transistor. Proceedings of the IEEE Symposium on Circuits and Systems.

[19] Haykin S., Widrow B. (2003). Least-Mean-Square Adaptive Filters.

[20] Venturi S.H.K., Panahi I. Hybrid RLS-NLMS Algorithm for Real-Time Remote Active Noise Control Using Directional UltraSonic LoudSpeaker. Proceedings of the IEEE 40th Annual Conference of the Industrial Electronics Society.

[21] Sharma D., Kaur R. Improvement in Convergence Speed and Stability of Least Mean Square and Normalized Least Mean Square Algorithm. Proceedings of the IEEE 2nd International Conference on Computing for Sustainable Global Development.

[22] De la Cruz-Alejo J., Oliva-Moreno L.N. (2013). LMS Algorithm for Programming an Analogue Memory Cell. Int. J. Electron..

[23] Figueroa M., Bridges S., Hsu D., Diorio C. (2004). A 19.2 GOPS Mixed Filter with Floating-Gate Adaptation. IEEE J. Solid-State Circuits..

[24] Rodríguez-Andina J.J., Valdés-Peña M.D., Moure M.J. (2015). Advanced Features and Industrial Applications of FPGA. IEEE Trans. Ind. Informat..

[25] Yen C.T., Weng W.-D., Lin Y.T. (2004). FPGA Realization of a Neural-Network-Based Nonlinear Channel Equalizer. IEEE Trans. Ind. Electron..

[26] Bian T., Jiang Y., Jiang Z. (2015). Decentralized Adaptive Optimal Control of Large-Scale Systems with Application to Power Systems. IEEE Trans. Ind. Electron..

